# Supplementary material for: True random number generation using the spin crossover in LaCoO3
Source: Nat Commun. 2024 May 31;15:4656. doi: 10.1038/s41467-024-49149-5 (PMC11143320; doi:10.1038/s41467-024-49149-5)
Supplement: Supplementary file 1 — Supplementary information [file 41467_2024_49149_MOESM1_ESM.pdf]

SUPPLEMENTARY INFORMATION

True Random Number Generation using the Spin Crossover in $LaCoO_3$

Kyung Seok Woo[1,2,3], Alan Zhang[1], Allison Arabelo[4], Timothy D. Brown[1], Minseong Park[1,2], A. Alec Talin[1], Elliot J. Fuller[1], Ravindra Singh Bisht[5], Xiaofeng Qian[4], Raymundo Arroyave[4], Shriram Ramanathan[5], Luke Thomas[6], R. Stanley Williams[1,2,*], and Suhas Kumar[1,*]

[1] Sandia National Laboratories, Livermore, CA, USA

[2] Department of Electrical and Computer Engineering, Texas A&M University, College Station, TX, USA

[3] Advanced Light Source, Lawrence Berkeley National Laboratory, Berkeley, CA, USA

[4] Department of Materials Science and Engineering, Texas A&M University, College Station, TX, USA

[5] Department of Electrical and Computer Engineering, Rutgers, The State University of New Jersey, Piscataway, NJ, USA

[6] Applied Materials Inc., Santa Clara, CA, USA

[*]Correspondence: R.S.W. (rstanleywilliams@tamu.edu), S.K. (su1@alumni.stanford.edu)

Table of contents

- **Supplementary Figs. 1-9**
- **Supplementary Tables 1-2**
- **Supplementary Notes 1-3**
- **Supplementary References**

Supplementary Figures

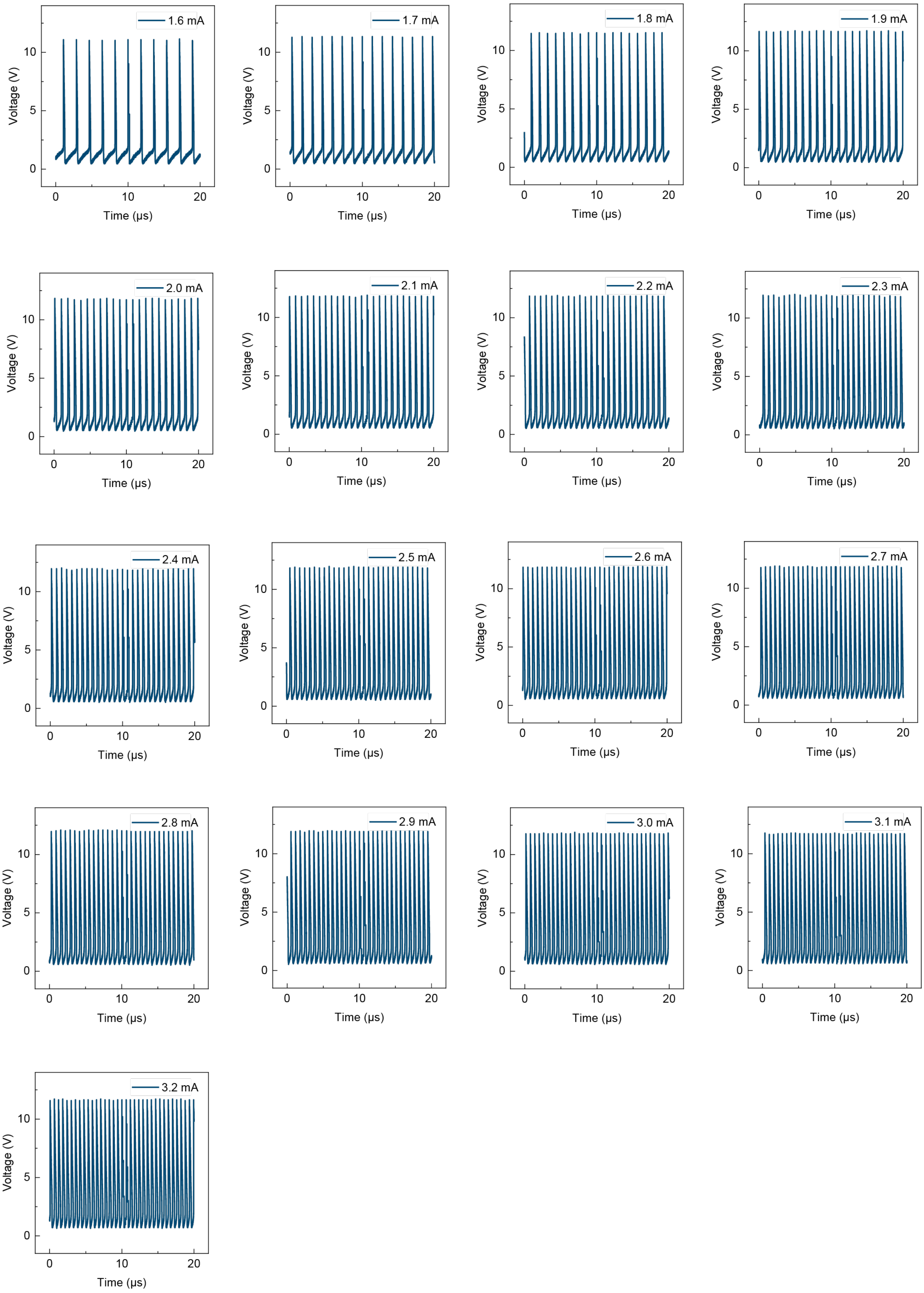

Supplementary Fig. 1 | Oscillations at different current levels.

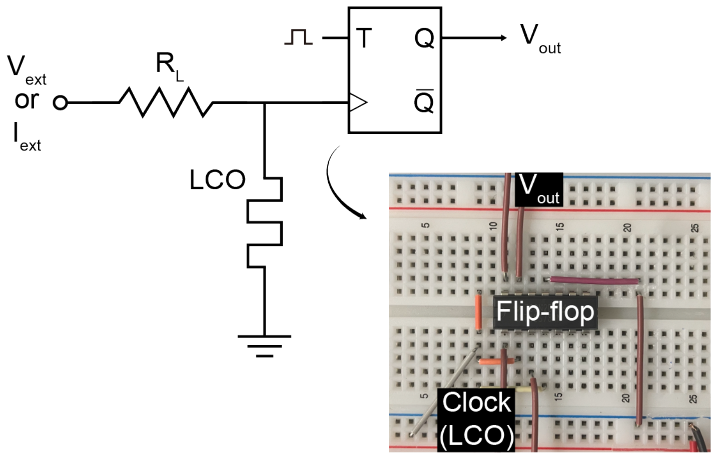

Supplementary Fig. 2 | Photograph of the proposed TRNG circuit.

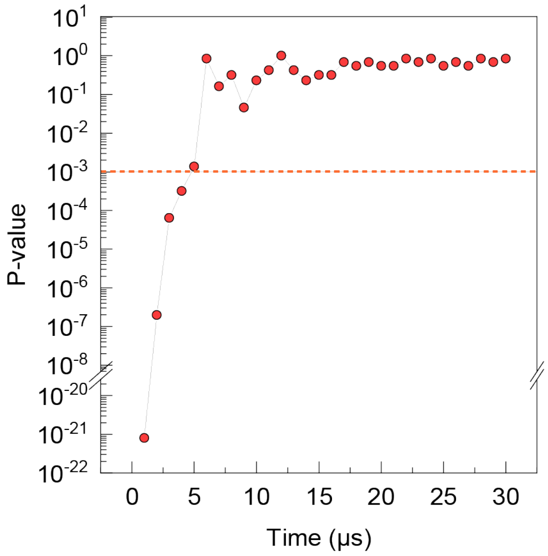

Supplementary Fig. 3 | Frequency (monobit) test results at different toggle (T) input pulse times. The bit generation rate can be increased by reducing T input pulse time, but there will be lesser number of oscillations (bit flipping) before producing the output bit. When the time was reduced to 5 μs, the resulting randomness significantly decreased and failed to pass the test at below 5 μs. However, there may be applications where data streams with limited randomness may be useful, wherein such a reduced T input pulse time would offer faster bit rates.

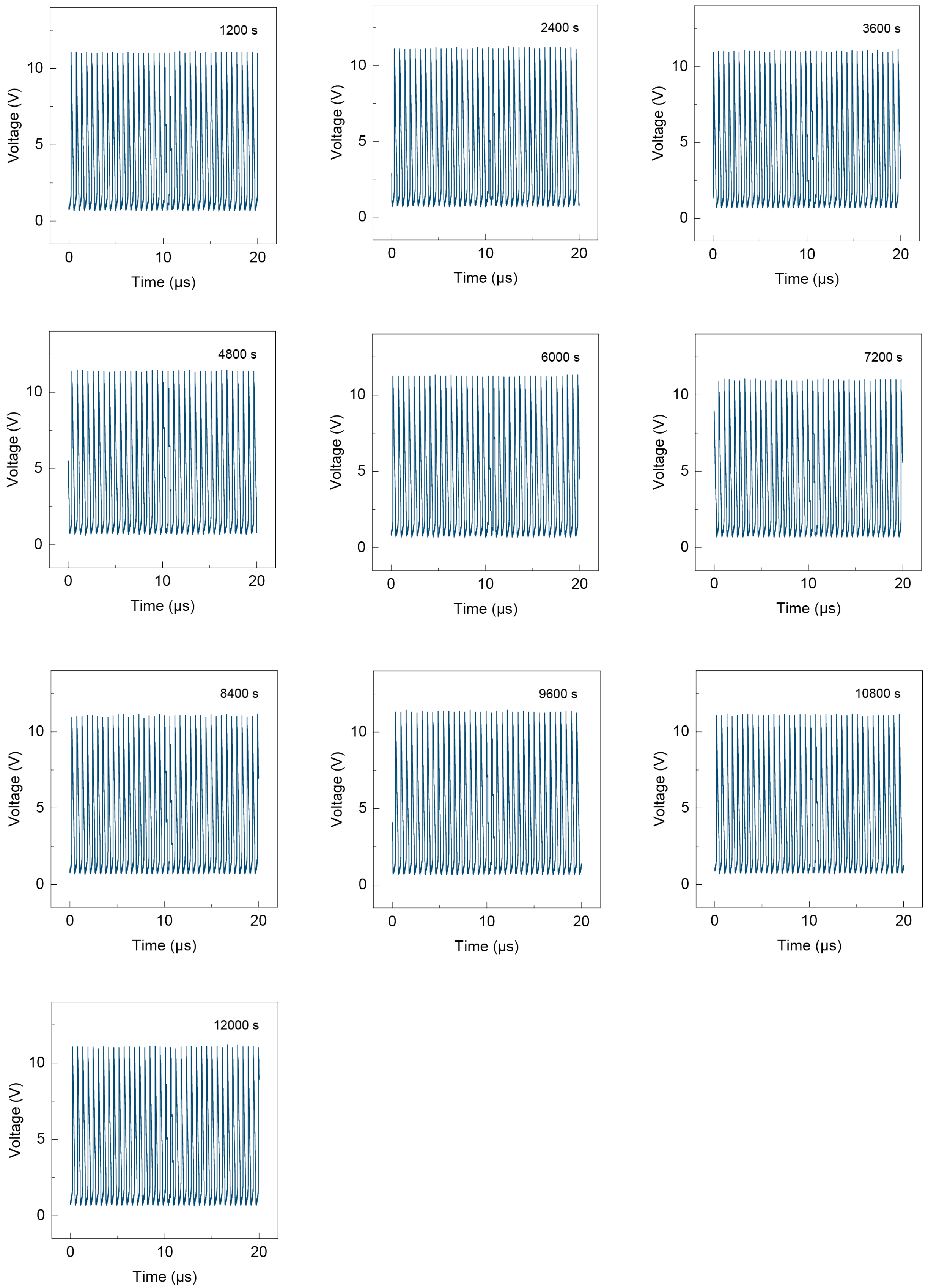

Supplementary Fig. 4 | Endurance test. The oscillations were monitored every 1200 seconds.

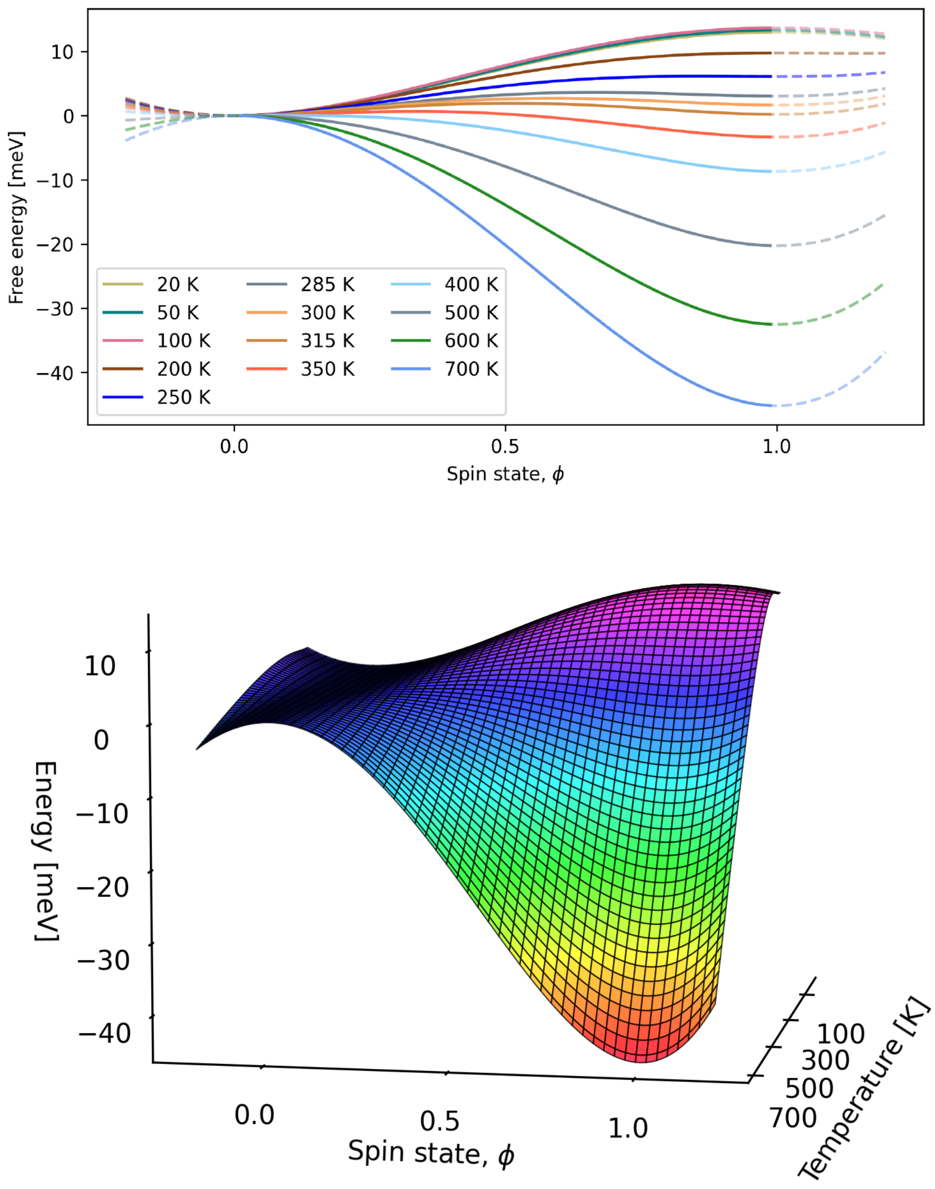

Supplementary Fig. 5 | Free energy landscapes as a function of temperature as determined from Eq. (S1).

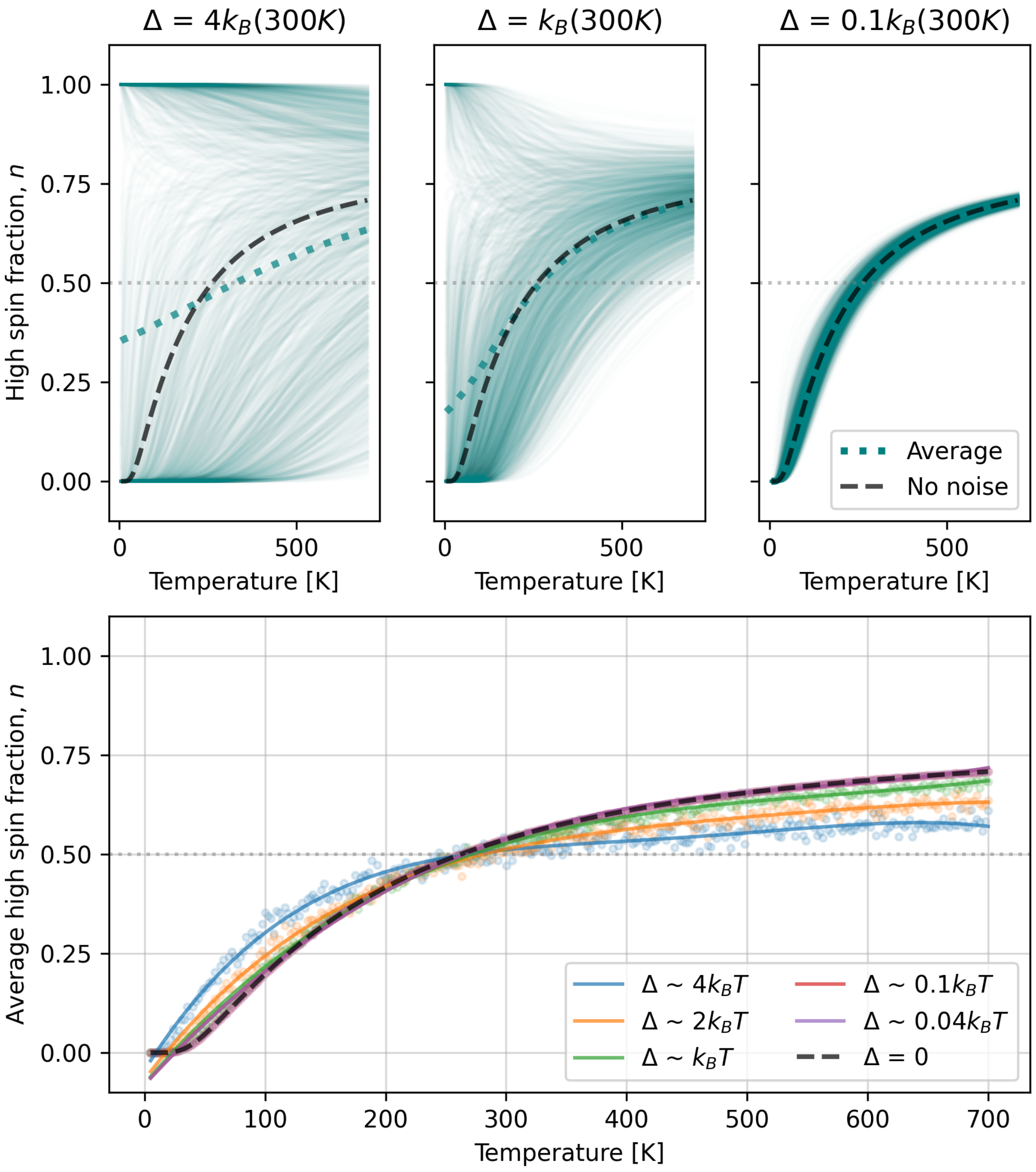

Supplementary Fig. 6 | Thermal distribution of the high spin fraction with temperature under the influence of thermal fluctuations, Δ, as determined from Eq. (S5) and Supplementary Table 2. A $\Delta = 0$ (dashed line) represents the thermodynamic limit, wherein no thermal fluctuations are present. The scattered points represent the average high spin fraction for 1500 samples, while the solid line represents the corresponding fitted line.

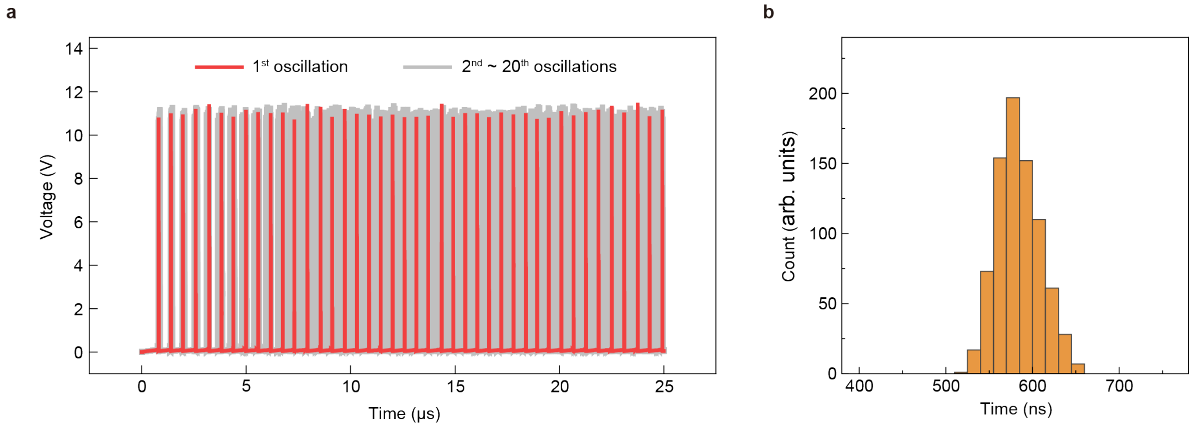

Supplementary Fig. 7 | a, Simulated 20 different oscillations. **b,** Distribution of time period in simulated oscillations.

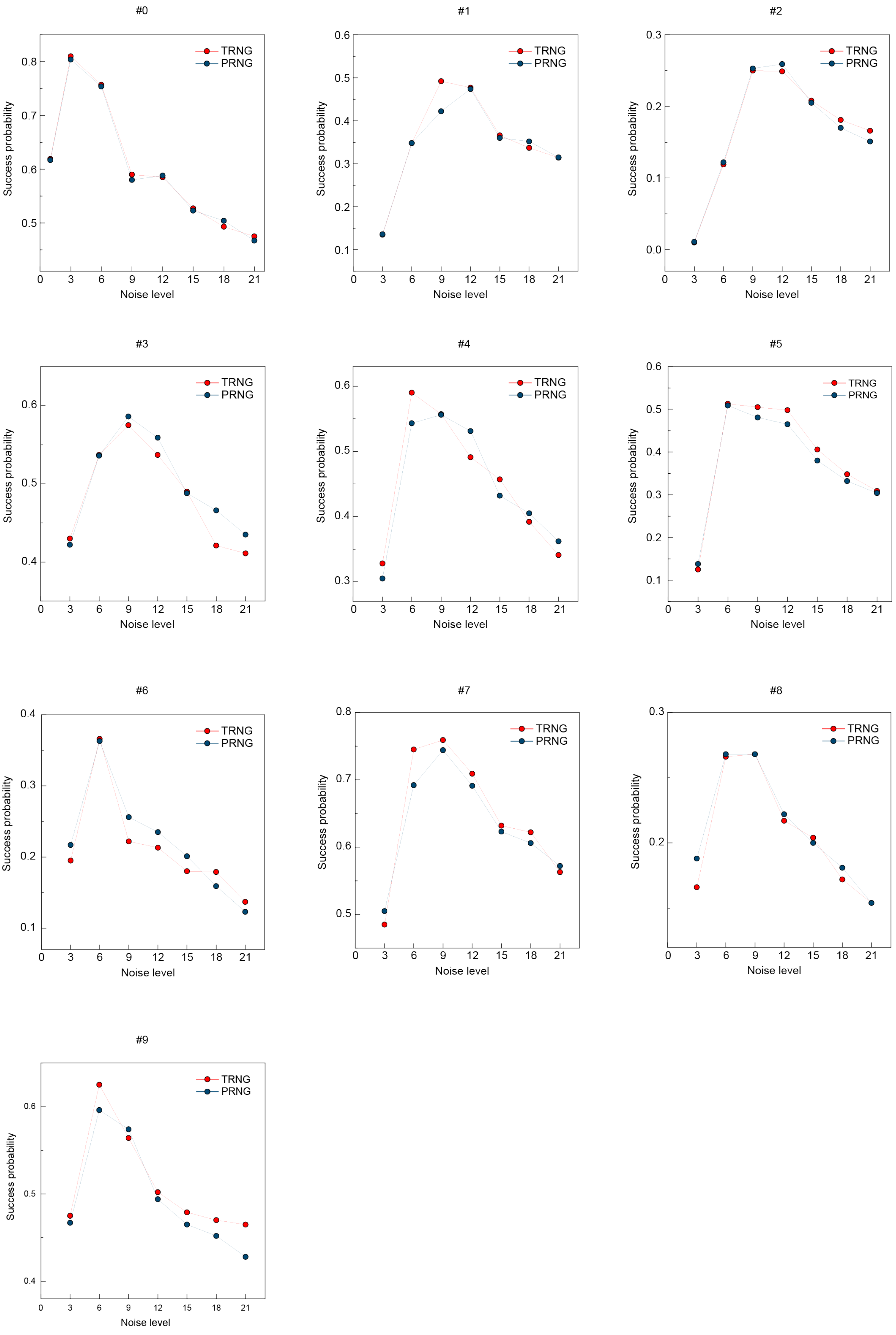

Supplementary Fig. 8 | Success probabilities for different problems.

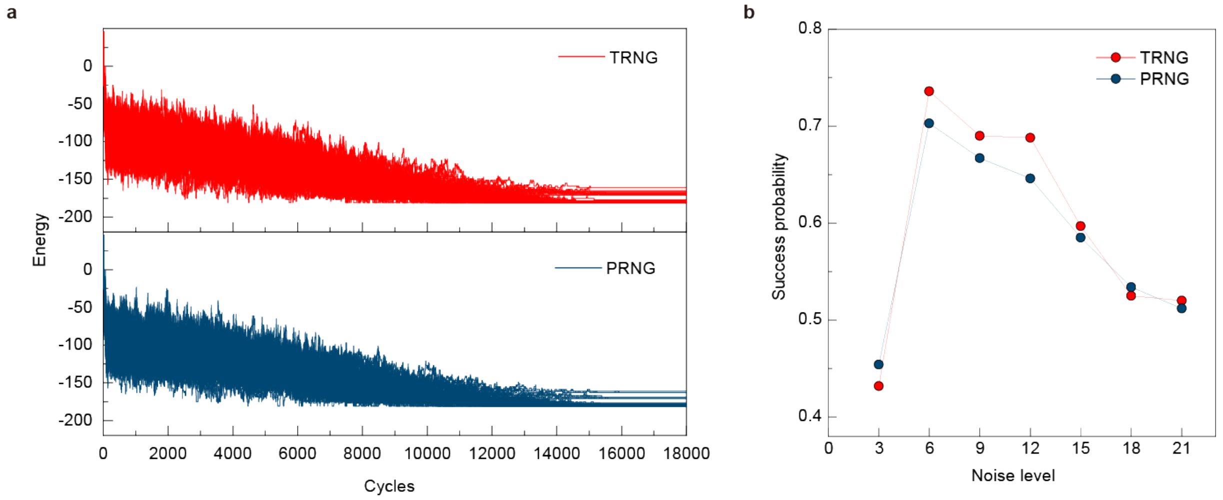

Supplementary Fig. 9 | a, Energy descent at 18000 iterations. **b,** Success probabilities at different noise levels.

Supplementary Tables

Supplementary Table 1. NIST randomness test results of the LCO-based TRNG.

	P-value	Pass rate	Pass/fail
**1. Frequency (Monobit) Test**	0.662957	80/80	Pass
**2. Frequency Test within a Block**	0.544515	80/80	Pass
**3. Runs Test**	0.060518	79/80	Pass
**4. Test for the Longest Run of Ones in a Block**	0.063835	80/80	Pass
**5. Binary Matrix Rank Test**	0.055594	80/80	Pass
**6. Discrete Fourier Transform**	0.051298	79/80	Pass
**7. Non-overlapping Template Matching Test**	0.049316	79/80	Pass
**8. Overlapping Template Matching Test**	0.101298	80/80	Pass
**9. Maurer's "Universal Statistical" Test**	0.004468	80/80	Pass
**10. Linear Complexity Test**	0.066100	80/80	Pass
**11. Serial Test**	0.719754	80/80	Pass
	0.578866	80/80	
**12. Approximate Entropy Test**	0.006355	79/80	Pass
**13. Cumulative Sums (Cusum) Test**	0.067257	80/80	Pass
	0.074361	80/80	
**14. Random Excursions Test**	0.013683	79/80	Pass
**15. Random Excursions Variant Test**	0.013699	79/80	Pass

Supplementary Table 2. Parameters used for the model.

Parameter	Value[4] (meV)	Parameter	Value (meV)	Parameter	Value[4]
ϵ_1	13	ε_1	-3.805	g_1	3
ϵ_2	35.5	ε_2	-3.866	g_2	5
ϵ_3	72.5	ε_3	-5.708	g_3	7

Supplementary Note 1. NIST randomness test.

The NIST randomness test is a set of 15 tests designed to assess the randomness of binary sequences, widely used in cryptographic applications to ensure the robustness and unpredictability of generated keys[1]. The results of these tests are evaluated using the *P*-value. The *P*-value is a statistical measure to which the observed data supports the null hypothesis. In the context of the NIST test, the null hypothesis is that a given binary sequence is random. Generally, a sequence is considered random if the *P*-value is greater than a predefined significance level, commonly set at 0.001. The bits collected from our LCO-based TRNG passed all the tests without any post-processing step, confirming its suitability for cryptographic applications.

Supplementary Note 2. Stochastic Thermodynamic Model.

Temperature-driven spin crossovers of LCO are influenced directly by the octahedral field acting on the metal ion, which consequently influences the electronic structure and energy levels. To describe the free energy landscape of the spin crossover phenomenon in LCO, we construct a free energy functional using a non-conserved order parameter (ϕ) accounting for the spin state and temperature (T) given as[2]:

$$\Delta f_{bulk}(\phi, T) = \int_V [(F_{HS} - F_{LS})\varphi(\phi) + \omega(n, T)g(\phi)]dV \quad \text{(S1)}$$

where F_{HS} and F_{LS} are the Helmholtz free energy for high spin (HS) and low spin (LS) states, respectively. The order parameter representing the spin states varies smoothly from $\phi = 0$ to $\phi = 1$ for the LS and HS configurations, respectively. Additionally, $\varphi(\phi) = \phi^2(3 - 2\phi)$ and $g(\phi) = \phi^2(1 - \phi)^2$ are interpolation functions, $\omega(n, T)$ is the barrier height of phase transformation, and n represents the population of the HS state. The Helmholtz free energy of LS and HS states can be obtained through $F = -k_B T \ln Z$ as:

$$F_{LS} = -k_B T \ln \left[\sum_i \exp\left(-\frac{\varepsilon_i n^2}{k_B T}\right)\right] \quad \text{(S2a)}$$

$$F_{HS} = -k_B T \ln \left[\sum_i g_i \exp\left(-\frac{\epsilon_i - 2\varepsilon_i n}{k_B T}\right)\right] \quad \text{(S2b)}$$

where k_B is Boltzmann constant, and the terms inside the brackets denote the partition function, Z, as the number of microstates that are thermally accessible to the electrons of the LS and HS states. Accordingly, g_i is the degeneracy of state i, and ϵ_i and ε_i are free parameters related to the energy difference between the LS and HS states and the lattice relaxation energy, respectively. The model essentially considers the symmetric relaxation of ligands[3] and three manifolds within the HS states of effective angular momentum J_i = 1, 2, 3 with degeneracy g_i = 3, 5, and 7, respectively[4]. The values of ε were approximated by choosing the set of parameters that improve the model relative to the model with $[\varepsilon_1, \varepsilon_2, \varepsilon_3] = [0, 0, 0]$. The HS population n is defined with respect to the probability of being in the HS state as[5,6]:

$$n = \frac{Z_{HS}}{Z} = \frac{Z_{HS}}{Z_{LS}+Z_{HS}} = \left(1 + \frac{Z_{LS}}{Z_{HS}}\right)^{-1} = \left[1 + \frac{\sum_i \exp(-\beta\varepsilon_i n^2)}{\sum_i g_i \exp[-\beta(\epsilon_i - 2\varepsilon_i n)]}\right]^{-1} \quad \text{(S3)}$$

where $\beta = (k_B T)^{-1}$. On the other hand, the SCO phenomenon is best described as a chemical equilibrium between two spin isomers (LS and HS, i.e., LS ⇌ HS[5,6]) rather than as a transition, that is, both spin states coexist as their relative populations shift with local variations in lattice distortion and thermal fluctuation. In this case, the activation energy E_a associated with this conversion can be obtained using the Arrhenius equation:

$$\ln K_{eq} = \ln \frac{n}{1-n} \approx \ln A - \frac{E_a}{k_B}\frac{1}{T} \quad \text{(S4)}$$

where K_{eq} is the equilibrium constant, and A is a preexponential constant. This activation energy is chosen to approximate the barrier $\omega(n, T)$. Moreover, acknowledging that the system's energy deviates from its average energy due to random thermal fluctuations, we modify the total energy in the partition function with a fluctuating component. We approximate this fluctuation in energy due to thermal noise as $\Delta \propto k_B T \Longrightarrow \Delta = m k_B T$, where m is a noise factor which adjusts the strength of the modeled fluctuation by directly varying the magnitude of Δ. The influence of random fluctuations can be observed by Gaussian sampling (i.e., 1,500 samples within $\Delta = \pm m k_B T$). Due to the barrier for transition being relatively low, the bistable LCO can be HS or LS at any temperature above 0 K. This is evident when one observes the evolution of the high-spin fraction when random thermal fluctuations are considered; as seen in Supplementary Fig. 6, this thermal evolution is stochastic rather than deterministic. As the magnitude of thermal fluctuations decreases, the evolution becomes more predictable and closely follows the thermodynamic limit ($\Delta = 0$, no noise).

$$n = \left[1 + \frac{\sum_i \exp(-\beta\varepsilon_i n^2 + \Delta)}{\sum_i g_i \exp(-\beta(\epsilon_i - 2\varepsilon_i n + \Delta))}\right]^{-1} \quad \text{(S5)}$$

Supplementary Note 3. Monte Carlo simulation.

The stochastic spiking response of LCO was carried out by Monte Carlo simulation using LT-spice. We employed a compact model of device geometry-based phase transition and enthalpy change[7]. The total device resistance can be expressed as:

$$R_{\mathrm{ch}}(u) = \frac{\rho_{\mathrm{ins}} L}{\pi r_{\mathrm{ch}}^2}\left[1 + \left(\frac{\rho_{\mathrm{ins}}}{\rho_{\mathrm{met}}} - 1\right) u^2\right]^{-1} \tag{S6}$$

where u is a state variable of the biphasic system, r_{ch} is conduction channel radius (20 nm), L is conduction channel length (100 nm), ρ_{ins} is insulating phase electrical resistivity (1.2×10^{-4} Ω·m), and ρ_{met} is metallic phase electrical resistivity (1.0×10^{-6} Ω·m). The thermal conductance of the insulating shell can be expressed as:

$$\Gamma_{\mathrm{th}}(u) = 2\pi L\kappa \left(\ln\frac{1}{u}\right)^{-1} \tag{S7}$$

where κ is thermal conductivity (10 $\mathrm{W{\cdot}m^{-1}K^{-1}}$). The derivative of total enthalpy change with respect to u can be expressed as:

$$\frac{\mathrm{d}\Delta H}{\mathrm{d}u} = \pi L r_{\mathrm{ch}}^2 [\hat{c}_p \Delta T \frac{1-u^2+2u^2\ln u}{2u(\ln u)^2} + 2\Delta\hat{h}_{\mathrm{tr}} u] \tag{S8}$$

where $\hat{c}_p$ is volumetric heat capacity (2.6×10^8 $\mathrm{J{\cdot}m^{-3}K^{-1}}$), ΔT is heating temperature (784 K), and $\Delta\hat{h}_{\mathrm{tr}}$ is volumetric enthalpy of transformation (1.6×10^8 $\mathrm{J{\cdot}m^{-3}}$). By combining Eq. (S7)-(S9), a first order nonlinear dynamical system can be derived as:

$$\frac{\mathrm{d}u}{\mathrm{d}t} = \left(\frac{\mathrm{d}\Delta H}{\mathrm{d}u}\right)^{-1} (R_{\mathrm{ch}}(u) i^2 - \Gamma_{\mathrm{th}}(u)\Delta T) \tag{S9}$$

where i is the current through the device[1]. The noise was added to κ by using a 'white' function with a scale factor of 0.1 under different time ranges for each repetition.

Supplementary References

1. Rukhin A. et al. NIST Special Publication 800-22 (2010).
2. Roy, A. M. Multiphase phase-field approach for solid-solid phase transformations via propagating interfacial phase in HMX. *J. Appl. Phys.* **129**, 025103 (2021).
3. Biernacki, S. W. Temperature-induced spin-state transitions in $LaCoO_3$: A two-level model. Phys. Rev. B **74**, 184420 (2006).
4. Tomiyasu, K. et al. Coulomb Correlations Intertwined with Spin and Orbital Excitations in $LaCoO_3$. *Phys. Rev. Lett.* **119**, 196402 (2017).
5. Bousseksou, A., Constant-Machado, H. & Varret, F. A Simple Ising-Like Model for Spin Conversion Including Molecular Vibrations. *J. Phys. I* **5**, 747–760 (1995).
6. Koudriavtsev, A. B. & Linert, W. Spin crossover - An unusual chemical equilibrium. *J. Struct. Chem.* **51**, 335–365 (2010).
7. Pickett, M. D. & Stanley Williams, R. Sub-100 fJ and sub-nanosecond thermally driven threshold switching in niobium oxide crosspoint nanodevices. *Nanotechnology* **23**, 215202 (2012).
